# Eosinophilic Digestive Disease (EDD) and Allergic Bronchial Asthma; Two Diseases or Expression of One Disease in Two Systems?

**DOI:** 10.1186/1824-7288-37-18

**Published:** 2011-04-16

**Authors:** Mostafa Yakoot

**Affiliations:** 1 Consultant Physician, Green Clinic and Research Centre

**Keywords:** Eosinophilic digestive disease, Bronchial asthma, Allergy

## Abstract

Eosinophilic digestive disease (EDD) includes a broad spectrum of clinical presentations due to eosinophilic inflammation involving anywhere from the esophagus to the rectum.

The heterogeneity in the clinical presentations of EDD is determined by the site and depth of eosinophilic infiltration. The sites of inflammation determine the nomenclature for EDD. The most well characterized of these, eosinophilic esophagitis (EE), eosinophilic gastroenteritis (EG), and eosinophilic colitis or enterocolitis. While the depth of esosinophilic infiltration through the three main layers (mucosa, musculosa and serosa) determines the prominent clinical manifestation. The recent advances in gastrointestinal endoscopy and the increasing awareness and diagnosis of EDD, in my viewpoint, can be of help to add to our understanding of the heterogeneous clinical syndrome under the broad title bronchial asthma.

Here I present my viewpoint that EDD and the allergic bronchial asthma can be regarded as two clinical expressions of one disease in two different but related anatomical systems.

## 

Recently, Eosinophilic Digestive Disease has become the focus of many published articles as well as discussions in scientific conferences[1,2]. The recent great advances in technology in the gastrointestinal endoscopy with more accessibility to highly versatile upper, lower as well as video-capsule endoscopes that can enable us to visualize and study every part of the alimentary canal, have revolutionized our understanding and researches in the digestive system to more clearly differentiate diseases with similar clinical pictures on basis of pathology and pathogenesis.

The common clinical presentations of abdominal pain, dyspepsia, bowel habits changes, nausea, vomiting, malabsorption and protein wasting can now be more accurately diagnosed on basis of pathology and pathogenesis. But in spite of that, my observation is that EDD is still under-diagnosed, as many cases of what I call mild intermittent attacks pass undiagnosed with empirical symptomatic treatment, or even misdiagnosed as gastroesophageal reflux disease (GERD) or irritable bowel syndrome (IBS),..etc.

Eosinophilic digestive disease (EDD) includes a broad spectrum of clinical presentations due to eosinophilic inflammation involving anywhere from the esophagus to the rectum [1,2].

The heterogeneity in the clinical presentations of EDD is determined by the site and depth of eosinophilic infiltration. The sites of inflammation determine the nomenclature for EDD. The most well characterized of these are eosinophilic esophagitis (EE), eosinophilic gastroenteritis (EG), and eosinophilic colitis or enterocolitis. While the depth of esosinophilic infiltration through the three main layers (mucosa, musculosa and serosa) determines the prominent clinical manifestation, for example, infiltration to the musculosa will be manifested by dysphagia in EE, or severe colic up to a picture of intestinal obstruction in EG and E enterocolitis. While subserosal and serosal affection will be manifested by ascites with abundant of eosinophils [3-6].

Tissue and blood eosinophilia (eosinophilic disorders) were broadly classified by Simons [7] according to the primary mechanism of pathogenesis into two main pathways. The intrinsic (eosinophils/or precursors) mutation mediated clonal expansion pathway; such as in some cases of leukemia and myeloproliferative diseases. The second pathway is the cytokine-mediated or extrinsic pathway. Interleukin-5 (IL-5) and to a lesser degree IL-3 and GM-CSF are the most critical cytokines mediating increased eosinophils differentiation, activation, and survival [7]. T helper-2 (TH2) inflammatory responses are induced by a variety of factors including allergic disorders, drug reaction with eosinophilia and in certain infections particularly by helminthes (worms). These responses are characterized by IgE antibody production and IL-5 induced eosinophilia [7].

Similar to allergic bronchial asthma, recent investigations support the allergen driven TH2 cytokine-mediated eosinophilic inflammation as the pathophysiologic mechanism of the EDD disease [1,2,4,8].

Whether the primary offender is an extrinsic one like an allergen taken by mouth or an intrinsic one like in case of parasitic infections, we are in favor to consider EDD as one disease.

The rationale behind this lies in our "unpublished observations" in 2 cases presenting with a full picture of EDD with abdominal pain, protein wasting, ascites with eosinophilia detected in peripheral blood, ascitic fluid as well as in biopsy tissues from lower endoscopy. The offending cause in these cases had been found to be parasitic infection by fascioliasis in the early migratory hepatic phase. The dramatic response to a course of systemic corticosteroids after the persistence of manifestations in spite of the treatment of fascioliasis, plus the typical picture of a multilayer eosinophilic enterocolitis, and the associated history of other atopic diseases have urged us to consider the term "intrinsic" rather than "secondary" EDD which hints to more heterogeneous pathogenetic factors for eosinophilia.

The recent advances in gastrointestinal endoscopy and the increasing awareness and diagnosis of EDD, in our mind, can be of help to add to our understanding of the heterogeneous clinical syndrome under the broad title bronchial asthma which remains a genuine medical mystery [9]. There is a need for more differentiation of cases on the basis of more homogeneous pathogenesis and pathology rather than the current definitions which are based mainly on the clinical manifestations leading to the inclusion of highly heterogeneous groups with many differing phenotypes, endotypes and response to treatment under one diagnosis [8-10].

There is no reason in my opinion, so far, to consider the allergic bronchial asthma or (the eosinophilic phenotype), and the eosinophilic bronchitis as two separate entities but we would rather explain this as in case of EDD on basis of the depth of eosinophilic and mast cell infiltration in the layers of the bronchial tissue whether to the layer of smooth muscle leading to asthma or just in mucosa and submucosa manifested as bronchitis [11,12].

Likewise the allergic rhinitis and allergic bronchial asthma can be regarded as one disease manifested in one system but in two different sites, like the case of EE and EG.

Further more with this view, the EDD and the allergic bronchial asthma can be regarded as two clinical expressions of one disease in two different but related anatomical systems, adding the fact that the embryological origin of the lower airway is a budding from the endoderm of the foregut.

It is obvious that the smooth muscle spasm leading to broncho-constriction, wheezes and air flow limitation in the respiratory system is manifested in the gastrointestinal tract as dysphagia when it affects the smooth muscle layer in the esophagus, or colic and intestinal obstruction when affecting the small intestine.

Even more, the circular strictures and longitudinal furrows seen in the endoscopic picture of the EE are much reminding us of the picture of bronchial smooth muscle spasm.

Also, the mucosal irritation with cough and sputum in the respiratory system is manifested in the EG by diarrhea with malabsorption and protein losing.

The documented association of the clinical picture of GERD with asthma and vice versa can be explained at least partly in some cases on basis of undiagnosed atopic EE associated with atopic asthma.

The subset of children who develops atopic or allergic asthma deserves to be differentiated as early as possible, through the personal or family history of atopy, combined if possible with testing for induced sputum eosinophilia or other immunological tests as advocated by Peter Sly and colleagues [13]. These subsets, according to our unpublished pragmatic real-world clinical study as well, can get much benefit from early interventions such as allergen avoidance, or treatment courses with a drug like ketotifen, which through not fully understood anti-allergic or cell stabilizing mechanisms, might have good impact on the natural evolution of the disease. Even in adults, this is the subset of patients I expect to be more responsive to controllers like corticosteroids, cromones and leukotriene modifiers.

The treatment of EDD, so far is similar to that of allergic bronchial asthma which includes avoidance of suspected allergens and systemic corticosteroids [2,4,14-16]. Leukotriene modifiers, and oral cromones had also been tried with some reports of success [17-20].

## Conclusion

With regard to this apparent strong interrelation between EDD and allergic bronchial asthma, not only in terms of similarity in pathogenesis, pathology and origin in embryology but also in drug therapy; might we be justified to simplify matters to use terms like allergic esophageal asthma or enteral asthma?

## Competing interests

The author declares that they have no competing interests.
